# Elevation of Intracellular Alpha-Ketoglutarate Levels Inhibits Osteoclastogenesis by Suppressing the NF-κB Signaling Pathway in a PHD1-Dependent Manner

**DOI:** 10.3390/nu15030701

**Published:** 2023-01-30

**Authors:** Junquan Tian, Xuetai Bao, Fan Yang, Xiongzhuo Tang, Qian Jiang, Yuying Li, Kang Yao, Yulong Yin

**Affiliations:** 1 Laboratory of Animal Nutritional Physiology and Metabolic Process, Key Laboratory of Agro-Ecological Processes in Subtropical Region, National Engineering Laboratory for Pollution Control and Waste Utilization in Livestock and Poultry Production, Institute of Subtropical Agriculture, Chinese Academy of Sciences, Changsha 410125, China; 2 University of Chinese Academy of Sciences, Beijing 100008, China; 3 College of Animal Science and Technology, Hunan Agricultural University, Changsha 410000, China; 4 Institute of Bast Fiber Crops, Chinese Academy of Agricultural Sciences, Changsha 410205, China

**Keywords:** alpha-ketoglutarate, osteoclast, PHD1, NF-κB

## Abstract

Age-related osteoporosis, a high-prevalence disease in the aged population, is generally attributed to the excessive activity of osteoclasts. Most approved drugs treat osteoporosis by inhibition of osteoclasts. Although in vivo studies have shown that alpha-ketoglutarate (AKG), an intermediate in the TCA cycle, can ameliorate age-related osteoporosis, the effects of AKG on osteoclastogenesis and the underlying mechanism of its action have not been studied yet. Here, we showed that the elevation of intracellular AKG levels by supplementing dimethyl AKG (DM-AKG, a cell-permeable derivative of AKG) inhibits the receptor activator of NF-κB ligand (RANKL)-induced osteoclasts differentiation from primary bone marrow-derived macrophages (BMMs) and RAW264.7 cells in vitro. We further found that DM-AKG treatment suppresses NF-κB signaling and oxidative phosphorylation (OXPHOS) during RANKL-induced osteoclastogenesis in RAW264.7 cells. Interestingly, dimethyl oxalylglycine (DMOG), an AKG competitive inhibitor of AKG-dependent prolyl hydroxylases (PHDs), antagonizes the suppression of the RANKL-activated NF-κB signaling pathway caused by DM-AKG treatment. Furthermore, blocked PHD1 expression (also known as EglN2), instead of PHD2 or PHD3, was confirmed to reverse the DM-AKG treatment-induced suppression of the RANKL-activated NF-κB signaling pathway. Accordingly, blocked PHD1 expression antagonized the inhibitory effects of DM-AKG on osteoclastogenesis. Together, our finding suggests that the elevation of intracellular AKG levels inhibits osteoclastogenesis by suppressing RANKL-activated NF-κB signaling in a PHD1-dependent manner, which may provide a novel nutritional strategy for osteoporosis treatment.

## 1. Introduction

Age-related osteoporosis, the most well-known bone disorder, has a high rate of prevalence in the aged population, resulting in a high risk of osteoporotic fracture, which increases the burden on the individual and on health care systems [1,2]. In recent years, studies have revealed that alpha-ketoglutarate (AKG) extends the lifespan of worms, drosophila, and mice, which makes the health care function of AKG during aging of significant concern [3,4,5,6,7]. Interestingly, the administration of AKG ameliorates age-related osteoporosis in aged mice and postmenopausal women [8,9]. Bone dynamic homeostasis is maintained by the balance between osteoclast-mediated resorption and osteoblast-mediated bone matrix formation in adults [10,11]. Osteoporosis patients generally show overactive osteoclasts activity, which facilitates bone resorption and leads to bone remodeling imbalance [12,13]. Therefore, dysregulated osteoclastogenesis is the main cause of osteoporosis and thus an essential therapeutic target. Most drugs approved by the national Food and Drug Administration (FDA) treat osteoporosis via the inhibition of osteoclasts [14]. However, the effects of AKG on osteoclasts are largely unknown.

AKG, an intermediate metabolite in the tricarboxylic acid (TCA) cycle, is generated from isocitrate via isocitrate dehydrogenase (IDH), and amino acids metabolism [15]. Beyond its traditional function as an intermediate metabolite, intracellular AKG is also the obligate cosubstrate of AKG-dependent dioxygenases, including prolyl hydroxylases (PHDs), histone demethylases (JHDMs), ten-eleven translocation family of 5-methylcytosine hydroxylases (TETs), and collagen prolyl-4-hydroxylase [15,16,17]. Because these AKG-dependent dioxygenases have a Km for AKG near cellular physiological concentrations, the intracellular AKG is supposed to be the key rate-limiting factor of these dioxygenases [17,18,19,20,21]. In recent years, accumulated evidence suggests that intracellular AKG plays a crucial role in regulating a wide range of cellular biological processes via AKG-dependent dioxygenases. For instance, a high level of intracellular AKG helps to maintain the pluripotency of naïve embryonic stem cells (ESCs) by promoting histone/DNA demethylation via stimulating the activity of AKG-dependent JHDMs and TETs [18]. In contrast, the elevation of intracellular AKG levels accelerates the differentiation of primed pluripotent stem cells (PSCs), by inducing global histone and DNA demethylation through JHDMs and TETs [19]. Notably, changes in intracellular AKG levels orchestrate macrophage polarization towards the M1 and M2 phenotype of macrophages through related AKG-dependent dioxygenases and AKG-mediated metabolic reprogramming [20]. However, how regulating intracellular AKG levels affects osteoclast differentiation remains unclear.

Osteoclasts are specialized cells derived from the monocyte/macrophage haematopoietic lineage. The receptor activator of nuclear factor κB ligand (RANKL) and macrophage colony-stimulating factor (M-CSF) jointly orchestrates osteoclast differentiation and maturation [13]. Primary bone marrow-derived macrophages (BMMs) and RAW264.7 cells, as homogenous cell populations of macrophage lineage, are commonly used as osteoclast precursors to mimic the differentiation of osteoclasts under the stimulation of RANKL in vitro [13,21]. Activated RANK signaling activates downstream diverse signaling pathways, including NF-κB signaling, AKT signaling, and MAPK signaling [13,22]. Many studies have revealed that the downregulation of the RANK signaling-activated signaling pathway, especially NF-κB signaling, is a proven therapeutic method for the treatment of age-related osteoporosis [23].

Given that AKG, a membrane-impermeable molecule, can only be transported into cells by active transportation through Na+/dicarboxylate co-transporter (NaDCs), the addition of exogenous AKG fails to directly alter intracellular AKG levels [24,25,26]. In contrast, dimethyl AKG (DM-AKG), an esterified variant of AKG, can indeed freely cross the phospholipid bilayer of the cytomembrane to reach cytosolic esterases that release AKG and hence promote intracellular AKG accumulation. Therefore, in previous studies, DM-AKG is widely used to increase intracellular AKG levels by adding DM-AKG to the medium [24]. In the present study, we found that DM-AKG inhibits RANKL-induced osteoclast differentiation in BMMs and RAW264.7 cells. Mechanistically, the elevation of intracellular AKG levels suppressed RANKL-stimulated activation of NF-κB signaling in a PHD1-dependent manner, which may provide a nutritional target for osteoporosis control in the aged population.

## 2. Materials and Methods

### 2.1. Regents

Alpha-ketoglutaric acid (AKG) disodium salt, dimethyl alpha-ketoglutarate (DM-AKG), and dimethyl oxalylglycine (DMOG) were purchased from Sigma-Aldrich. Recombinant murine RANKL and M-CSF were purchased from Peprotech (Rocky Hill, NJ, USA). Antibodies for phospho-IKKα/β, IKKβ, phospho-IκBα, IκBα, NF-κB (P65), histone H3, and β-actin were purchased from Cell Signaling Technology (Beverly, MA, USA). Antibodies for PHD1, PHD2, and PHD3 were purchased from Abcam (Billerica, MA, USA). The RAW264.7 cell line was purchased from the American Type Culture Collection (ATCC, Manassas, VA, USA). Dulbecco’s Modified Eagle’s Medium (DMEM), α-minimum essential medium (α-MEM), fetal bovine serum (FBS), penicillin, and streptomycin were purchased from Gibco (Rockville, MD, USA).

### 2.2. Animals

Four-week-old female wild-type C57BL/6 mice were acquired from the SALC Laboratory Animal Central in Changsha, China. All mice were kept in a pathogen-free mouse colony with a 12 h light/dark cycle, maintained at a temperature of 25 ± 2 °C and relative humidity of 45–60%, and supplied with standard laboratory food and water ad libitum. All animal protocols were approved by the Laboratory Animal Ethical Commission of the Institute of Subtropical Agriculture (Permit No. ISA2020037 for the mice experiment), Chinese Academy of Sciences.

### 2.3. Cell Culture

Cells were cultured in DMEM or α-MEM supplemented with 10% (*v*/*v*) FBS, penicillin (100 units/mL), and streptomycin (100 ug/mL) at 37 °C in a humidified 5% CO_2_ atmosphere.

### 2.4. Cell Viability Assays

To evaluate the effect of DM-AKG on cell viability, RAW264.7 cells (3 × 10^4^ cells/cm^2^) or bone marrow cells (6 × 10^4^ cells/cm^2^) were cultured with or without DM-AKG in 96-well plates for 24 h, 48 h, or 72 h, respectively. Because DM-AKG is consumable, we changed the medium with fresh DM-AKG every 24 h, according to previous reports [19]. Cell viability was ascertained by utilizing the Cell Counting Kit-8 (CCK-8, Dojindo, Japan), in accordance with the supplier’s instructions.

### 2.5. Osteoclastogenesis from RAW264.7 Cells

For osteoclast induction, RAW264.7 cells (5 × 10^4^ cells/cm^2^) were treated with 100 ng/mL RANKL and cultured in the DMEM with 10 % FBS. After 3 days, TRAP staining was conducted using a TRAP staining Kit (Solarbio, Beijing, China), according to the manufacturer’s instructions. The TRAP activity was determined by following the instructions of the TRAP activity Kit (Beyotime, Shanghai, China). ImageJ software (1.52P version) was utilized to measure the surface areas of the osteoclast.

### 2.6. Osteoclastogenesis from Bone Marrow-Derived Macrophages (BMMs)

To acquire primary mouse BMMs, the bone marrow of femoral bones from 8-week-old C57BL6 mice were flushed with serum-free α-MEM. The cells derived from the bone marrow were cultured in growth media α-MEM supplemented with 10% FBS. Nonadherent cells were retrieved and incubated in a medium that was supplemented with 50 ng/mL of M-CSF after a period of 24 h. The adherent cells were put to use as BMMs after a 48-h duration. For osteoclast induction, the BMMs were seeded with the density of 1 × 10^5^ cells/per well in a 24-well plate and cultured in the medium with 100 ng/mL RANKL and 50 ng/mL M-CSF for 4 days. Osteoclastogenesis was determined through TRAP staining by using the TRAP staining Kit (Solarbio, Beijing, China), following the manufacturer’s guidelines. The TRAP activity was evaluated by following the manufacturer’s instructions with the TRAP activity Kit (Beyotime, Shanghai, China).

### 2.7. Mitochondrial Oxidative Phosphorylation Assays

The effects of DM-AKG on mitochondrial oxidative phosphorylation in RANKL-stimulated RAW264.7 cells were assessed by the XF24 Analyser and XF Cell Mito Stress Test Kit (Seahorse Biosciences, North Billerica, MA, USA), as described previously [27]. Briefly, following the treatment, the culture medium was substituted with the designated test medium. During the test, the oligomycin (0.5 μM), carbonyl cyanide 4-(trifluoromethoxy), phenylhydrazone (FCCP, 1.0 μM), and rotenone/antimycin A (1.0 μM) were sequentially injected into the test medium. Measurement of basic respiration, maximal respiration capacity, ATP production, and proton leak were obtained through the probes. The total protein content of the cells was measured in order to normalize the final data. Seahorse XF Report Generator software (Wave, Agilent) was utilized to analyze the measurement results.

### 2.8. Cell Transfection

RAW264.7 cells were seeded in 24-well plates at a density of 1 × 10^5^ cells/well. Reaching 80% confluency, the cells were transfected with 500 ng plasmids employing Lipofectamine3000 (Thermo Fisher Scientific, CA, USA), in accordance with the manufacturer’s instructions.

### 2.9. Double Nicking CRISPR-Cas9

Double nicking CRISPR-Cas9 was conducted according to our previous study [28]. In brief, guide RNAs were developed utilizing the CRISPR design tool (http://crispr.mit.edu, accessed on 20 October 2020) and then cloned into the BbsI-digested plasmids (pSpCas9n) with the complete guide RNA scaffold. After transfection, in order to obtain a stably transfected polyclonal cell line, the cells were incubated with 5 μg/mL puromycin in DMEM/F12 medium for 4 days. In addition, the stably transfected polyclonal cell line that expresses green fluorescent protein (GFP) was verified by fluorescence microscopy. Monoclonal cell lines were isolated by carrying out serial dilutions. The knock-out efficiency of target proteins was determined by western blot (WB).

### 2.10. Real-Time Quantitative PCR (RT-qPCR)

RAW264.7 cells (2 × 10^5^ cells/well) were cultured in a 24-well plate. RNA was extracted from the sample using an E.Z.N.A.R Total RNA Kit I (Omega Bio-Tek, USA), following the manufacturer’s guidelines. Total RNA was measured using a Nanodrop ND-1000 (Wilmington, DE, USA). The amount of 1 μg of total RNA was converted into cDNA using a reverse transcription system from TAKARA (TAKARA BIO) according to the manufacturer’s instructions. RT-qPCR was performed using TB GreenTM (TAKARA BIO) according to the manufacturer’s instructions, and the results were detected using a LightCycler480^®^Ⅱmachine. β-Actin was employed as an endogenous reference to normalize the quantifies of target mRNA by using the formula 2^–ΔΔCT^ (ΔΔCT = ΔCT sample—ΔCT control). All primer sequences are listed in Supplementary Table S1.

### 2.11. Western Blot Analysis

Western blot (WB) experiments were conducted as previously described [29]. In brief, utilizing the Nuclear and Cytoplasmic Protein Extraction Kit (Thermo Fisher Scientific, USA), cytoplasmic and nuclear proteins were isolated. Protein concentrations were measured by a BCA kit (Beyotime Biotechnology, Shanghai, China) followed by adjusting them to a uniform concentration. The protein samples were mixed with a 5× SDS-PAGE sample loading buffer (Beyotime Biotechnology, Shanghai, China), followed by a 95 °C water bath for 5 min. Equal amounts of samples were separated on 10% SDS-poly-acrylamide gels. The proteins were then transferred onto polyvinylidene difluoride (PVDF) membranes and blocked with 5% bovine serum albumin for 1 h. The blots were allowed to incubate with the relevant primary antibodies for 12 h at a temperature of 4℃. Following the washing process, secondary antibodies were allowed to incubate for one hour at 25 °C before the blots were developed using Odyssey Infrared Imaging (Bio-Rad).

### 2.12. Immunofluorescence

RAW264.7 cells were seeded on sterilized coverslips in 6-well plates. At room temperature, the cells on coverslips were fixed with 4% paraformaldehyde for 30 min, followed by three washes with PBS in two-minute intervals. The cells on coverslips were then exposed to 5% (vol/vol) Triton-X 100 for a period of 5 min. Following a threefold two-minute PBS wash and a one-hour block with 5% defatted milk solution, the cells were incubated with primary antibodies of target proteins in a humidity chamber overnight at 4 °C. The cells on slides underwent three cycles of 2 min washings with PBST prior to incubation with the secondary antibody. Finally, after the nuclei were stained with DAPI (1:1000, Beyotime Biotechnology, Shanghai, China), the coverslips with cells were transferred to slices and sealed with a fluorescence anti-quenching agent (Beyotime Biotechnology, Shanghai, China). The images were obtained using a confocal laser scanning microscope (Zeiss, Germany). The fluorescence ratio of nuclear protein to cytoplasmic protein was measured by ImageJ software (1.52P version).

### 2.13. Statistical Analyses

Data, presented as means ± SEM, were statistically analyzed using the SPSS 22.0 software. Shapiro-Wilk was used to analyze whether the data conforms to a normal distribution. If the data conforms to a normal distribution, T-test or one-way ANOVA was used to analyze the data, and Duncan’s multiple range test was used for multiple comparisons. If the data fails to meet a normal distribution, statistical comparisons were analyzed by Mann-Whitney test. The number of samples in the experiment is indicated in each figure legend. * *p* < 0.05 and ** *p* < 0.01 were considered to be statistically significant. All data are representative of at least three independent experiments.

## 3. Results

### 3.1. The Elevation of Intracellular AKG Levels Inhibits Osteoclastogenesis

**Figure 1:**
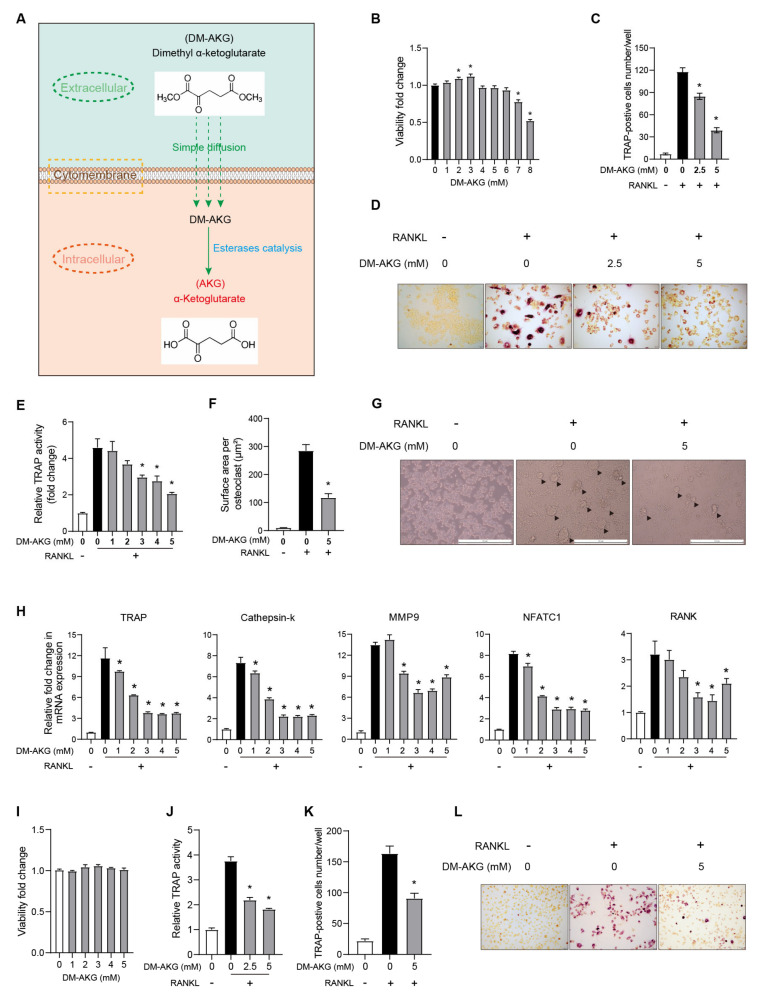
DM-AKG inhibits RANKL-induced osteoclast differentiation from RAW264.7 cells and BMMs. (**A**) Schematic illustration of DM-AKG entering cells and further being cleaved to AKG. (**B**) Effects of DM-AKG on the viability of RAW264.7 cells (*n* = 7). (**C**) Quantification of osteoclasts by TRAP staining, (**D**) representative photomicrographs of TRAP-positive cells, (**E**) TRAP activity, and (**H**) mRNA levels of osteoblast marker genes in RAW264.7 cells treated with DM-AKG and RANKL for 3 d (*n* = 3). (**F**) Surface area of multinucleated osteoblast (*n* = 3) and (**G**) representative images of multinucleated osteoclast under various conditions for 5 days. The osteoclasts were indicated by black triangles. (**I**) Effects of DM-AKG on the viability of BMMs (*n* = 4). (**J**) TRAP activity (*n* = 3), (**K**) TRAP-positive cell number (*n* = 3), and (**L**) representative images of osteoblast differentiated from BMMs for 4 d. All data were presented as means ± SEM. *, *p* < 0.05.

Dimethyl-AKG (DM-AKG), a widely used cell-permeable derivative of AKG, easily increases intracellular AKG levels [24] (Figure 1A). To determine a concentration range where DM-AKG does not affect the viability of RAW264.7 cells, the cells were exposed to various concentrations of DM-AKG (0, 1, 3, 4, 5, 6, 7, and 8 mM) for 72 h. As shown in Figure 1B, DM-AKG at 7 and 8 mM inhibited cell viability. We thus used 5 mM as a maximal concentration of DM-AKG in the following experiments. To examine the effect of DM-AKG on RANKL-induced osteoclastogenesis, we incubated RANKL-stimulated RAW264.7 cells with various concentrations of DM-AKG and then evaluated the osteoclast formation. DM-AKG treatment reduced the number of TRAP-positive cells (Figure 1C,D) and TRAP activity (Figure 1E) in a dose-dependent manner. In addition, DM-AKG (5 mM) treatment reduced the size of multinucleated osteoclasts at the late stage of RANKL-induced osteoclastogenesis (Figure 1F,G). RT-qPCR results showed that DM-AKG inhibited RANKL-induced mRNA levels of TRAP, Cathepsin-k, MMP9, NFATC1, and RANK (marker genes of osteoclast) in a dose-dependent manner (Figure 1H). Therefore, these results suggest that the elevation of intracellular AKG levels by DM-AKG supplementation inhibits RANKL-induced osteoclast differentiation from RAW264.7 cells.

We further explored the effects of DM-AKG treatment on RANKL-induced osteoclast differentiation from primary BMMs. As shown in Figure 1I, the cell viability of BMMs was not negatively affected by DM-AKG (0, 1, 3, 4, and 5 μM) supplementation for 72 h. The activity of TRAP in the cells was determined during osteoclast differentiation, the results of which showed that DM-AKG inhibited RANKL-induced the increase of TRAP activity in a dose-dependent manner (Figure 1J). Furthermore, DM-AKG (5 mM) treatment reduced the number of TRAP-positive cells (Figure 1K,L). Collectively, these results indicated that the elevation of intracellular AKG levels inhibits RANKL-induced osteoclast differentiation from BMMs.

Although direct AKG supplementation to the medium cannot directly alter intracellular AKG levels, studies reported that AKG can activate its cell membrane receptor GPR99 (also known as OXGR1) to regulate cellular functions [30,31,32]. We next assessed the effects of the addition of AKG disodium salt to the medium on RANKL-induced osteoclastogenesis from RAW264.7 cells. Our results showed that an extracellular AKG concentration greater than 15 mM significantly inhibited cell viability (Figure S1A,B). Further, RANKL-stimulated RAW264.7 cells were incubated with series concentrations (0.1 mM, 1 mM, and 10 mM) of AKG. TRAP staining and TRAP activity results showed that this extracellular AKG treatment did not affect the RANKL-induced increase of TRAP-positive cells (Figure S1C,E) and TRAP activity (Figure S1D). RT-qPCR results showed that this AKG treatment did not affect mRNA levels of *TRAP*, *Cathepsin-k*, *MMP9*, *NFATC1*, and *RANK* (marker genes of osteoclast marker) (Figure S1F). Collectively, these results suggest that extracellular AKG failed to inhibit RANKL-induced osteoclastogenesis from RAW264.7 cells.

### 3.2. OXPHOS of the RANKL-Stimulated RAW264.7 Cells Is Attenuated by DM-AKG Treatment

**Figure 2:**
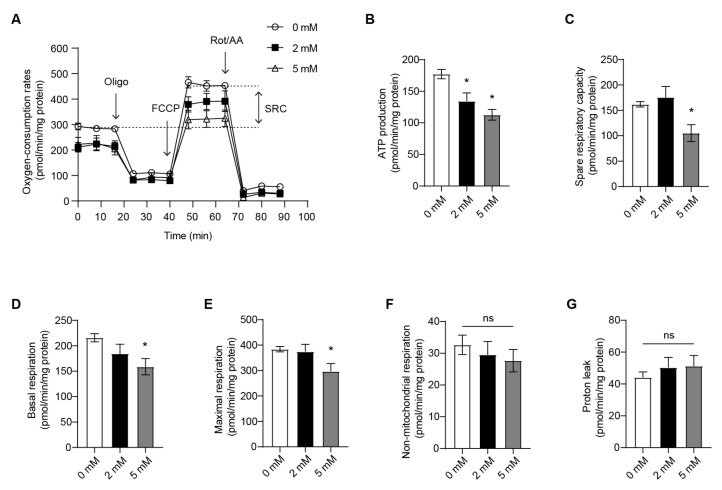
DM-AKG attenuated OXPHOS in RANKL-stimulated RAW264.7 cells. (**A**) Oxygen-consumption rates, (**B**) ATP production, (**C**) spare respiratory capacity (SRC), (**D**) basal respiration, (**E**) maximal respiration, (**F**) non-mitochondrial respiration, and (**G**) proton leak in RAW264.7 cells treated with RANKL and DM-AKG (0, 2 or 5 mM) for 12 h before treatment with oligomycin (oligo), carbonyl cyanide-p-trifluoromethoxyphenylhydrazone (FCCP), and rotenone plus antimycin A (Rot/AA) (*n* = 4). All data were presented as means ± SEM. *, *p* < 0.05. Ns, no significance.

Osteoclast differentiation from the progenitor induces metabolic remodeling to promote oxygen consumption and enhances mitochondrial oxidative phosphorylation (OXPHOS) to cover high ATP demands [33,34]. AKG is a key intermediate in the TCA cycle, which implies that the elevation of intracellular AKG levels may disturb the energy metabolism of the cells. Therefore, we investigated the effects of DM-AKG treatment on the OXPHOS of RAW264.7 cells during RANKL-induced osteoclastogenesis. The oxygen-consumption rates (OCR) of the cells were monitored using a Seahorse extracellular flux analyzer. ATP production, spare respiratory capacity (SRC), basal respiration, maximal respiration, non-mitochondrial respiration, and proton leak were calculated by the general scheme of OCR (Figure 2A). The results indicated that DM-AKG (5 mM) treatment significantly suppressed ATP production, SRC, basal respiration, and maximal respiration in RAW264.7 cells during RANKL stimulation (Figure 2B–E). There is no difference in non-mitochondrial respiration and proton leak among various groups (Figure 2F,G). These results suggest that DM-AKG treatment attenuates OXPHOS during RANKL-induced osteoclastogenesis.

### 3.3. The Elevation of Intracellular AKG Levels Suppresses NF-κB Signaling during RANKL-Induced Osteoclast Differentiation

**Figure 3:**
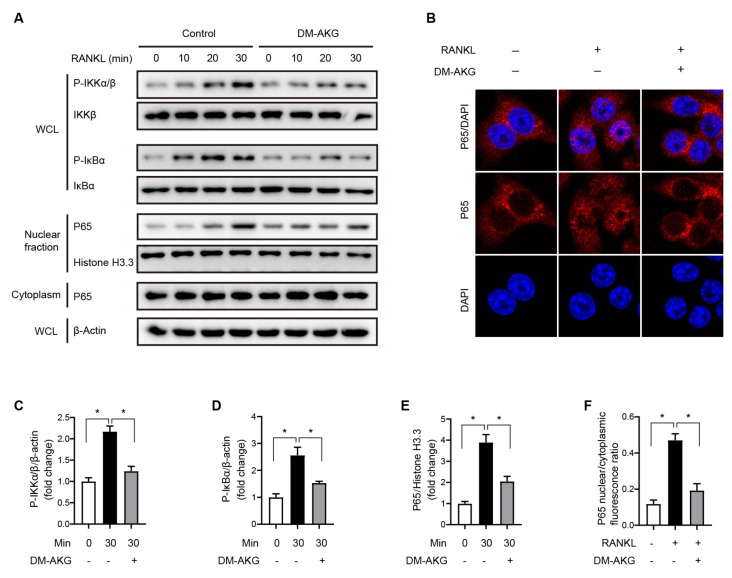
DM-AKG inhibits RANKL-induced NF-κB activation in RAW264.7 cells. (**A**) Immunoblot analysis of nuclear and cytoplasm P65, phosphorylated (P-) IKKα/β, IKKβ, phosphorylated (P-) IκBα, and IκBα in RAW264.7 cells incubated with or without 5 mM DM-AKG for 3 h and then stimulated with RANKL. (*n* = 3). WCL, whole-cell lysate. (**B**) Representative images of immunofluorescence for P65 incubated with or without 5 mM DM-AKG for 3 h and then stimulated with or without RANKL for 30 min. Quantification of immunoblot of (**C**) nuclear P65, (**D**) P-IKKα/β, and (**E**) P-IκBα incubated with or without 5 mM DM-AKG for 3 h and then stimulated with RANKL for 30 min (*n* = 3). (**F**) Quantitative immunofluorescence results of P65 nuclear translation (*n* = 3). All data were presented as means ± SEM. *, *p* < 0.05.

To determine the molecular mechanism involved in the inhibitory effect of DM-AKG on RANKL-induced osteoclast differentiation, we explored whether DM-AKG suppresses RANKL-stimulated NF-κB signaling activation, which is critical for osteoclast differentiation [13]. The binding of RANKL to its receptor, RANK, leads to the phosphorylation of IκB kinase-α/β (IKKα/β) leading to the phosphorylation of IκBα and the liberation of NF-κB, and the eventual nuclear translocation of the P65-P50 heterodimer [13]. In the present study, RAW264.7 cells incubated with or without DM-AKG for 3 h were stimulated with RANKL stimulated for various durations (0, 10, 20, and 30 min). WB analysis revealed that the pretreatment of DM-AKG significantly inhibited RANKL-stimulated IKKα/β phosphorylation (Figure 3A,C). Consistent with impaired IKKα/β phosphorylation, its downstream phosphorylation of IκBα was significantly suppressed by DM-AKG (5 mM) pretreatment (Figure 3A,D). Furthermore, pretreatment with DM-AKG significantly suppressed RANKL-stimulated the nuclear translocation of P65 (Figure 3A,E). Accordingly, immunofluorescence results showed that pretreatment with DM-AKG (5 mM) significantly inhibits the RANKL-stimulated nuclear translocation of P65 (Figure 3B,F). Thus, these results demonstrated that the elevation of intracellular AKG levels inhibits the RANKL-stimulated activation of the NF-κB signaling pathway during osteoclast differentiation.

### 3.4. DMOG Antagonizes the Inhibition Effects of DM-AKG on NF-κB Signaling Activation during Osteoclast Differentiation

**Figure 4:**
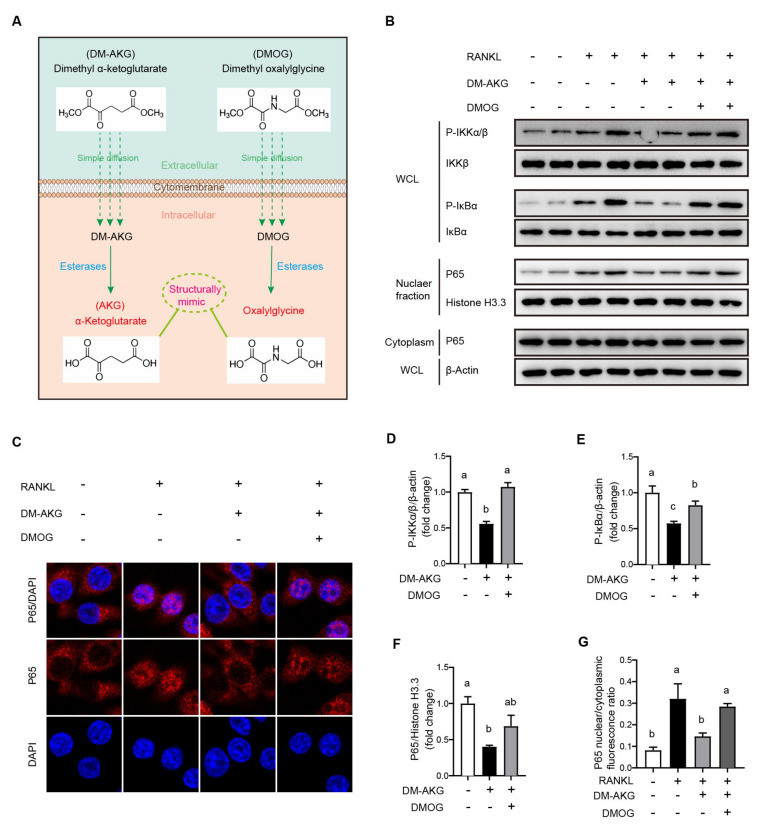
DMOG antagonizes the inhibitory effects of DM-AKG on the NF-κB signaling pathway during osteoclast differentiation. (**A**) Schematic illustration of DMOG entering cells and further being cleaved to oxalylglycine to antagonize AKG. (**B**) Immunoblot analysis of nuclear and cytoplasm NF-κB P65, phosphorylated (P-) IKKα/β, IKKβ, phosphorylated (P-) IκBα, and IκBα in RAW264.7 incubated with DMOG (5 mM) for 2 h and then co-incubated with DM-AKG for 3 h (5 mM), followed by RANKL stimulation for 30 min. WCL, whole-cell lysate. (**C**) Representative images of immunofluorescence for P65 in RAW264.7 cells incubated with DMOG (5 mM) for 2 h and then co-incubated with DM-AKG for 3 h (5 mM) followed by RANKL stimulation for 30 min. WCL, whole-cell lysate. Quantification of immunoblot of (**D**) nuclear P65, (**E**) P-IKKα/β, and (**F**) P-IκBα (*n* = 3). (**G**) Quantitative immunofluorescence results of P65 nuclear translation (*n* = 3). All data were presented as means ± SEM. No matching letters between the two groups point to a significant difference.

To elucidate whether AKG-dependent dioxygenases are involved in the regulation of DM-AKG treatment to the RANKL-stimulated NF-κB pathway, we assessed the effects of the AKG-dependent dioxygenase inhibitor dimethyl oxalylglycine (DMOG) on the DM-AKG interfered NF-κB signaling pathway during osteoclast differentiation. The membrane-permeable ester DMOG can also enter the cells by simple fission and increases intracellular oxalylglycine levels (Figure 4A). Oxalylglycine is a structural analogue of AKG except that the –CH_2_– of No. 3 on the carbon frame is replaced by –NH–. Intracellular oxalylglycine can bind to and function as a competitive inhibitor of AKG-dependent dioxygenases [16]. RAW264.7 cells were incubated with DMOG (5 mM) for 2 h and then co-incubated with DM-AKG for 3 h (5 mM) followed by RANKL stimulation for 30 min. WB analysis results showed that DMOG reversed the inhibitory effects of DM-AKG on RANKL-stimulated IKKα/β phosphorylation (Figure 4B,D). Furthermore, DMOG reversed the inhibitory effects of DM-AKG on RANKL-stimulated IκBα phosphorylation (Figure 4B,E). Accordingly, DMOG reversed the inhibitory effects of DM-AKG on the RANKL-stimulated nuclear translocation of P65 (Figure 4B,F). In agreement with WB analysis results, immunofluorescence results showed that DMOG reversed the inhibitory effects of DM-AKG on RANKL-stimulated nuclear translocation of P65 (Figure 4C,G). Collectively, these results suggest that the elevation of intracellular AKG levels suppresses the NF-κB pathway of RANKL-stimulated cells by activating AKG-dependent dioxygenase.

### 3.5. PHD1 Deficiency Reverses the Inhibitory Effects of DM-AKG Treatment on NF-κB Signaling during Osteoclast Differentiation

**Figure 5:**
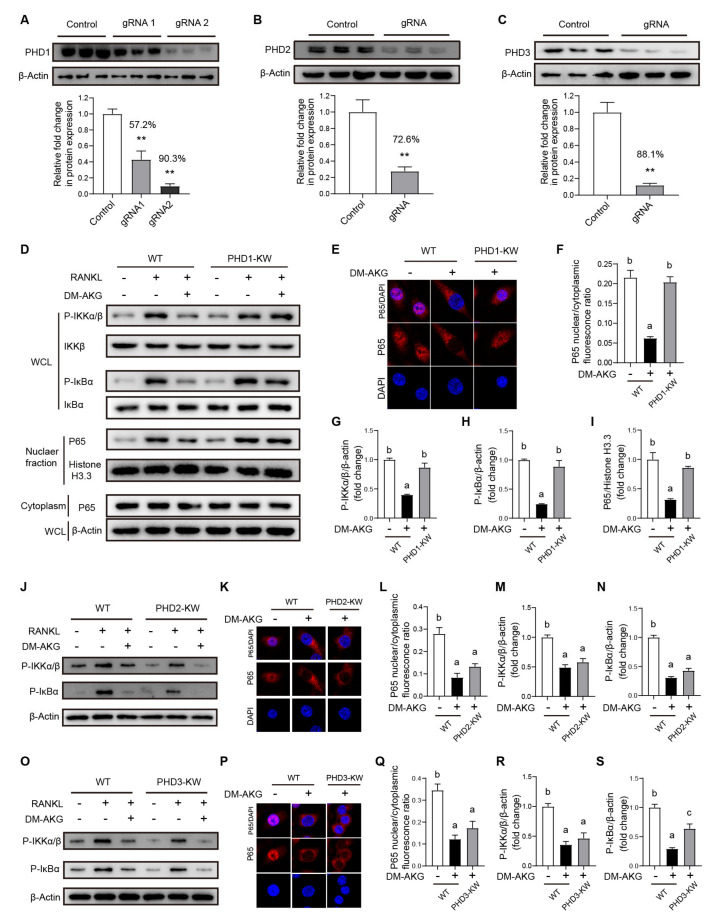
Blocked PHD1 expression reverses the inhibitory effects of DM-AKG on NF-κB signaling pathway during osteoclast differentiation. (**A**) The knock-out efficiency of PHD1 in two monoclonal cell lines determined by WB (*n* = 3). The knock-out efficiency of (**B**) PHD2 and (**C**) PHD3 in monoclonal RAW264.7 cell line determined by WB (*n* = 3). (**D**) Immunoblot analysis of nuclear and cytoplasm P65, P-IKKα/β, IKKβ, P-IκBα, and IκBα in WT and PHD1 deficiency cells treated with or without DM-AKG (5 mM) for 3 h and then stimulated with or without RANKL for 30 min. Representative images of immunofluorescence for P65 (**E**) and quantitative immunofluorescence results of P65 nuclear translation (**F**) in WT and PHD1 deficiency cells treated with or without DM-AKG (5 mM) for 3 h and then stimulated with RANKL for 30 min (*n* = 3). Quantitative results of immunoblot of nuclear P65 (**G**), P-IKKα/β (**H**), and (**I**) P-IκBα in WT and PHD1-deficiency RAW264.7 cells (*n* = 3). (**J**) Immunoblot analysis of P-IKKα/β, IKKβ, P-IκBα, and IκBα in WT and PHD2 deficiency cells treated with or without DM-AKG (5 mM) for 3 h and then stimulated with or without RANKL for 30 min. Representative images of immunofluorescence for P65 (**K**) and quantitative immunofluorescence results of P65 nuclear translation (**L**) in WT and PHD2-deficiency RAW264.7 cells treated with or without DM-AKG (5 mM) for 3 h and then stimulated with RANKL for 30 min (*n* = 3). Quantitative results of immunoblot of P-IKKα/β (**M**), and P-IκBα (**N**) in WT and PHD2 deficiency cells (*n* = 3). (**O**) Immunoblot analysis of P-IKKα/β, IKKβ, P-IκBα, and IκBα in WT and PHD3 deficiency cells treated with or without DM-AKG (5 mM) for 3 h and then stimulated with or without RANKL for 30 min. Representative images of immunofluorescence for P65 (**P**) and quantitative immunofluorescence results of P65 nuclear translation (**Q**) in WT and PHD3 deficiency cells treated with or without DM-AKG (5 mM) for 3 h and then stimulated with RANKL for 30 min. Quantitative results of immunoblot of P-IKKα/β (**R**), and P-IκBα (**S**) in WT and PHD3 deficiency cells (*n* = 3). All data were presented as means ± SEM. No matching letters between the two groups point to a significant difference. **, *p* < 0.01.

To further determine whether the elevation of intracellular AKG levels suppresses the RANKL-activated NF-κB pathway via AKG-dependent dioxygenase PHD1, PHD2, or PHD3, we respectively tested the effects of PHD1, PHD2, or PHD3 deficiency on the DM-AKG treatment-induced inhibition of NF-κB signaling during osteoclast differentiation in RAW264.7 cells. We respectively obtained PHD1-, PHD2-, and PHD3-deficiency RAW264.7 monoclonal cell lines using CRISPR-Case9. The WB analysis results showed that the PHD1, PHD2, and PHD3 were effectively knocked down by 90.3%, 72.6%, and 88.1%, respectively (Figure 5A–C).

The PHD1, PHD2, or PHD3-deficiency RAW264.7 cells were incubated with DM-AKG (5 mM) for 3 h followed by RANKL stimulation for 30 min. WB analysis results showed that blocked PHD1 expression reversed the inhibitory effects of DM-AKG on RANKL-stimulated IKKα/β and its downstream IκBα phosphorylation (Figure 5D,G,H) and further reversed the inhibitory effects of DM-AKG on the RANKL-stimulated nuclear translocation of P65 (Figure 5D,I). Accordingly, immunofluorescence results showed that blocked PHD1 expression reversed the inhibitory effects of DM-AKG on the RANKL-stimulated nuclear translocation of P65 (Figure 5E,F). However, neither blocked PHD2 nor PHD3 expression affected the roles of DM-AKG on RANKL-stimulated NF-κB signaling during osteoclast differentiation. Collectively, these results demonstrated that the elevation of intracellular AKG levels suppresses the RANKL-stimulated activation of the NF-κB pathway via PHD1, instead of PHD2 or PHD3, during osteoclast differentiation.

### 3.6. PHD1 Deficiency Antagonizes the Inhibitory Effects of DM-AKG on Osteoclast Differentiation

**Figure 6:**
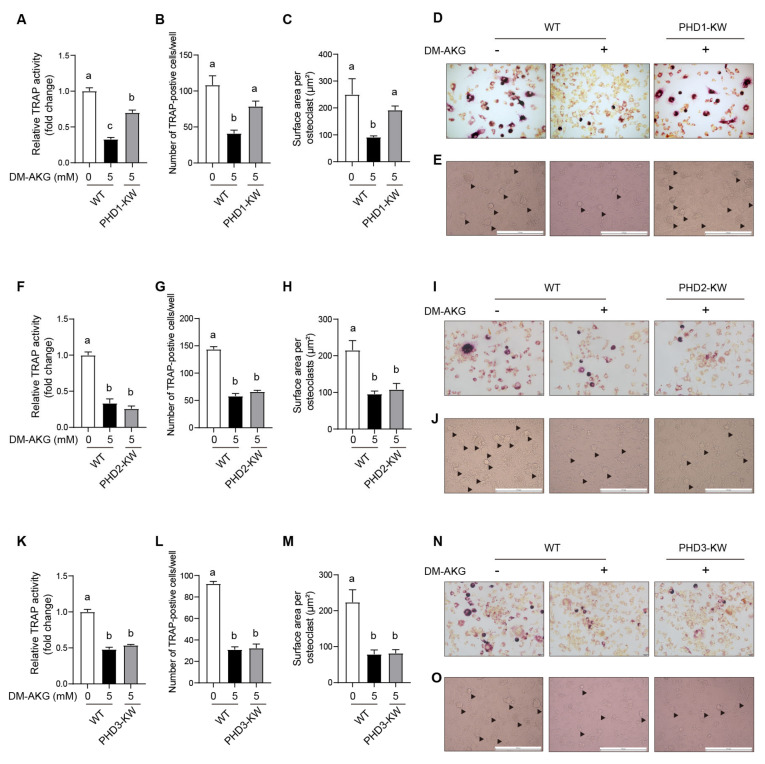
PHD1 deficiency antagonizes the effects of DM-AKG on inhibiting osteoclast differentiation. (**A**) TRAP activity, (**B**) number of TRAP-positive cells, and (**D**) representative photographs of TRAP activity staining in WT and PHD1-deficiency RAW264.7 cells treated with 5 mM DM-AKG and RANKL for 3 days (*n* = 3). (**C**) Surface area of multinucleated osteoblasts, and (**E**) representative images of multinucleated osteoclasts from WT and PHD1-deficiency RAW264.7 cells treated with 5 mM DM-AKG and RANKL for 5 days (*n* = 3). (**F**) TRAP activity, (**G**) number of TRAP-positive cells, and (**I**) representative photomicrographs of TRAP activity staining in WT and PHD2-deficiency RAW264.7 cells treated with 5 mM DM-AKG and RANKL for 3 days (*n* = 3). (**H**) Surface area of multinucleated osteoblasts, and (**J**) representative images of multinucleated osteoclasts from WT and PHD2-deficiency RAW264.7 cells treated with 5 mM DM-AKG and RANKL for 5 days (*n* = 3). (**K**) TRAP activity, (**L**) number of TRAP-positive cells, and (**N**) representative photomicrographs of TRAP activity staining WT and PHD3-deficiency RAW264.7 cells treated with 5 mM DM-AKG and RANKL for 3 days (*n* = 3). (**M**) Surface area of multinucleated osteoblasts, and (**O**) representative images of multinucleated osteoclasts from WT and PHD3-deficiency RAW264.7 cells treated with 5 mM DM-AKG and RANKL for 5 days (*n* = 3). The osteoclasts were indicated by black triangles. All data were presented as means ± SEM. No matching letters between the two groups point to a significant difference.

We next explored whether blocked PHD1 expression antagonizes the inhibitory effects of DM-AKG on RANKL-induced osteoclast differentiation. PHD1, PHD2, or PHD3-deficiency RAW264.7 cells were treated with DM-AKG (5 mM) and RANKL to induce osteoclast differentiation. TRAP activity results showed that blocked PHD1 expression alleviated the inhibitory effects of DM-AKG on RANKL-stimulated TRAP activity (Figure 6A). Accordingly, blocked PHD1 expression reversed the DM-AKG treatment-induced decrease in the number of TRAP-positive cells (Figure 6B,D) and the surface area of multinucleated osteoclast (Figure 6C,E) during RANKL-induced osteoclast differentiation. However, neither blocked PHD2 (Figure 6F–J) nor PHD3 (Figure 6K–O) expression affected DM-AKG’s role in TRAP activity, TRAP-positive cell, or the surface area of multinucleated osteoclasts during RANKL-induced osteoclast differentiation. These results demonstrated that the elevation of intracellular AKG levels inhibits RANKL-induced osteoclast differentiation through PHD1 instead of PHD2 or PHD3.

## 4. Discussion

Age-related osteoporosis has a high prevalence rate in the aged population and is usually caused by excessive osteoclast activity. Recent studies reported the potential of AKG in ameliorating age-related osteoporosis and extending the lifespan of worms, drosophila, and mice. Moreover, accumulated evidence suggests that intracellular AKG, as an activator for AKG-dependent dioxygenases, participates in the regulation of a wide range of cellular biological processes via related AKG-dependent dioxygenases. To the best of our knowledge, this is the first in vitro study to explore the influence of AKG on osteoclastogenesis and the underlying mechanism of its action. We discovered that the elevation of intracellular AKG levels inhibits RANKL-induced osteoclastogenesis by suppressing NF-κB signaling in a PHD1-dependent manner.

Age-related osteoporosis is closely related to excessive osteoclast activity [12,13]. Although in vivo studies have shown that the administration of AKG can ameliorate osteoporosis in aged mice, postmenopausal women, ovariectomized rats, and orchidectomized rats [8,9,35,36,37], the direct effects of AKG on osteoclastogenesis remains unclear. In addition, accumulated evidence suggests that AKG can regulate intracellular AKG-dependent dioxygenase activity or activate its membrane receptor, GPR99, to regulate some cellular biological activities [18,19,20,30,31,32,38]. In this study, we respectively investigated the effects of the addition of AKG disodium salt and DM-AKG to the medium on the RANKL-induced osteoclast differentiation in vitro. Our results demonstrate that adding 5 mM DM-AKG to the medium significantly inhibited RANKL-stimulated osteoclastogenesis in RAW264.7 cells and BMMs. However, 0.1 mM, 1 mM, and 10 mM AKG supplementation to the medium failed to affect RANKL-stimulated osteoclastogenesis from RAW264.7 cells. Because an earlier study demonstrated that IC50 of human GPR99 is 69 ± 11 μM [39], the AKG concentrations up to 10 mM are sufficient to activate GPR99 on the cell membrane if GPR99 is expressed in osteoclasts. Thus, we assume that extracellular AKG has no effect on osteoclastogenesis. The transmembrane transport rate of AKG depends not only on extracellular AKG concentration but also on the number of NaDCs, the transporter protein of AKG, on the osteoclast’s membrane. Furthermore, NaDCs are tissue-specific and only highly expressed in some tissues, such as the kidney, small intestine, and placenta [40]. These factors may lead to the low transmembrane transport efficiency of AKG. In addition, intracellular AKG is easily consumed. We therefore speculate that the addition of 10 mM AKG disodium to the medium did not promote sufficient intracellular AKG accumulation to affect osteoclastogenesis. In contrast, numerous studies have proved that DM-AKG can cross the cell membrane in a simple diffusion manner and effectively increase intracellular AKG levels. Thus, we conclude that the elevation of intracellular AKG levels inhibits osteoclast osteoclastogenesis in vitro.

Changes in energy metabolism, such as enhanced OXPHOS and glycolysis, are generally observed during osteoclast differentiation [33,34]. AKG is a key intermediate in the TCA cycle that provides electrons for OXPHOS [15], which prompted us to investigate the effects of DM-AKG treatment on OXPHOS during osteoclastogenesis. Theoretically, it seems that DM-AKG treatment can enhance OXPHOS. Unexpectedly, our results demonstrated that OXPHOS of the RANKL-stimulated cells is attenuated by DM-AKG treatment during osteoclastogenesis. Therefore, the DM-AKG treatment-weakened OXPHOS may serve as a potential mechanism by which intracellular AKG infusion inhibits RANKL-induced osteoclastogenesis. Given that cells during osteoclastogenesis generally show high OXPHOS levels, the decreased OXPHOS of RANKL-stimulated cells in DM-AKG groups may otherwise be the results caused by different levels of osteoclast differentiation.

The NF-κB signaling pathway plays a key role in osteoclast formation and bone resorption [23]. Of note, an earlier study revealed that intracellular AKG can suppress hypoxia-induced NF-κB signaling activity [41]. A recent study found that DM-AKG treatment promotes endotoxin tolerance by attenuating the NF-κB signaling pathway in glutamine-deprived bone-marrow-derived macrophages [20]. Therefore, we assessed the effects of DM-AKG on NF-κB signaling during RANKL-induced osteoclastogenesis in RAW264.7 cells. Our results suggest that the elevation of intracellular AKG levels by supplementing DM-AKG inhibits RANKL-activated NF-κB signaling during osteoclastogenesis.

Several studies have shown that the elevation of intracellular AKG levels can stimulate the activity of multiple AKG-dependent dioxygenases to modulate the pluripotency of ESCs, macrophage polarization, and the senescence of bone marrow mesenchymal stem cells [8,18,19,20]. We therefore hypothesized that DM-AKG supplementation suppresses RANKL-activated NF-κB signaling by stimulating the activity of AKG-dependent dioxygenases. Numerous studies have shown that DMOG, which binds to and functions as a competitive inhibitor of AKG-dependent dioxygenases, can antagonize the increased intracellular AKG level-induced changes in various cellular biological processes [16]. Of note, our results showed that DMOG reverses the inhibitory effects of DM-AKG on the RANKL-activated NF-κB signaling pathway. Therefore, we conclude that DM-AKG treatment suppresses the NF-κB signaling pathway by augmenting the activity of AKG-dependent dioxygenases during RANKL-induced osteoclastogenesis. This result also prompted us to further determine which AKG-dependent dioxygenase mediates the interesting action of DM-AKG in osteoclastogenesis.

An earlier study demonstrated that increasing PHD activity can suppress the hypoxia-induced activation of NF-κB signaling [41]. Notably, a recent study showed that intracellular AKG suppresses the NF-κB signaling pathway by augmenting PHD activity, thereby impairing proinflammatory responses in M1 macrophages [20]. Thus, in the present study, we first explored whether AKG-dependent prolyl hydroxylases, PHDs, mediate the inhibitory effects of DM-AKG on the RANKL-activated NF-κB signaling pathway. Interestingly, our results demonstrated that blocked PHD1 expression, rather than PHD2 or PHD3, reversed the suppression of the RANKL-activated NF-κB signaling pathway caused by DM-AKG treatment. Previous studies have shown that IKKβ, within the activation loop, contains an evolutionarily conservative LxxLAP consensus motif for the hydroxylation by PHDs [41,42]. PHD1 has been reported to hydroxylate the proline residue within the LxxLAP motif of IKKβ (P191) [41]. The hydroxylation of P191 suppresses IKKβ activation by forming the steric hindrance of the phosphorylation of nearby residues S177 and S181 within the activation loop [20,41]. In the present study, DM-AKG treatment suppresses the RANKL-induced phosphorylation of IKKα/β, while both DMOG treatment and blocked PHD1 expression reverse the inhibitory effects of DM-AKG on the RANKL-induced phosphorylation of IKKα/β. Therefore, we conclude that DM-AKG treatment suppresses the RANKL-induced activation of the NF-κB signaling pathway by augmenting PHD1 activity.

Accordingly, our results further demonstrated that among the three PHDs, only blocked PHD1 expression efficiently antagonized the inhibitory effects of DM-AKG on RANKL-induced osteoclastogenesis. Thus, we further conclude that the elevation of intracellular AKG levels by the addition of DM-AKG to the medium inhibits RANKL-induced osteoclastogenesis by suppressing the NF-κB signaling pathway in a PHD1-dependendent manner. Interestingly, studies have reported that the serum AKG levels in human and mice gradually decreases during aging [8,38,43]. Our findings consistently support the idea that low serum levels of AKG in the aged population may contribute to osteoclastogenesis and age-related osteoporosis. Further in vivo research is still needed to clarify this hypothesis.

## 5. Conclusions

**Figure 7:**
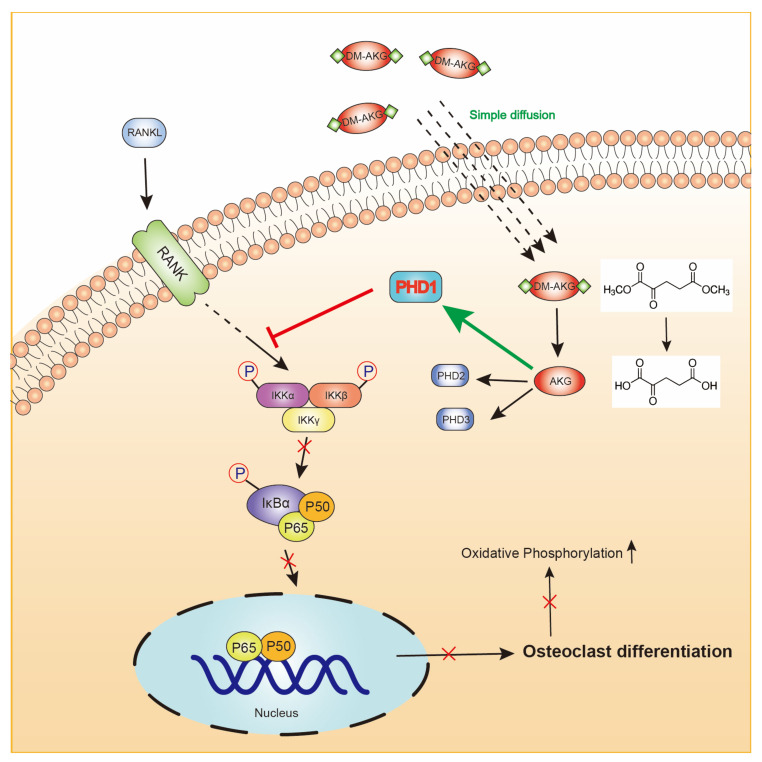
The proposed molecular mechanism underlying the elevation of intracellular AKG levels inhibits the RANKL-induced osteoclast differentiation.

In summary, our study demonstrated that DM-AKG treatment suppresses RANKL-induced osteoclastogenesis in BMMs and RAW264.7 cells in vitro. Mechanistically, the elevation of intracellular AKG levels suppresses the NF-κB signaling pathway in a PHD1-dependent manner during RANKL-induced osteoclastogenesis (Figure 7). Our findings may inspire new ideas for the treatment of age-related osteoporosis.

## Data Availability

Not applicable.

## Supplementary Materials

The following supporting information can be downloaded at: https://www.mdpi.com/article/10.3390/nu15030701/s1, Figure S1: Extracellular AKG failed to inhibit RANKL-induced osteoclast differentiation in RAW264.7 cells; Table S1: Primer sequence of the genes for RT-PCR in the study.

## References

[1] Vilaca T., Eastell R., Schini M. (2022). Osteoporosis in men. Lancet Diabetes Endocrinol..

[2] Compston J.E., McClung M.R., Leslie W.D. (2019). Osteoporosis. Lancet.

[3] Demidenko O., Barardo D., Budovskii V., Finnemore R., Palmer F.R., Kennedy B.K., Budovskaya Y.V. (2021). Rejuvant^®^, a potential life-extending compound formulation with alpha-ketoglutarate and vitamins, conferred an average 8 year reduction in biological aging, after an average of 7 months of use, in the TruAge DNA methylation test. Aging.

[4] Yang F., Zhou Z., Guo M., Zhou Z. (2022). The study of skin hydration, anti-wrinkles function improvement of anti-aging cream with alpha-ketoglutarate. J. Cosmet. Dermatol..

[5] Shahmirzadi A.A., Edgar D., Liao C.-Y., Hsu Y.-M., Lucanic M., Shahmirzadi A.A., Wiley C.D., Gan G., Kim D.E., Kasler H.G. (2020). Alpha-Ketoglutarate, an Endogenous Metabolite, Extends Lifespan and Compresses Morbidity in Aging Mice. Cell Metab..

[6] Chin R.M., Fu X., Pai M.Y., Vergnes L., Hwang H., Deng G., Diep S., Lomenick B., Meli V.S., Monsalve G.C. (2014). The metabolite α-ketoglutarate extends lifespan by inhibiting ATP synthase and TOR. Nature.

[7] Su Y., Wang T., Wu N., Li D., Fan X., Xu Z., Mishra S.K., Yang M. (2019). Alpha-ketoglutarate extends Drosophila lifespan by inhibiting mTOR and activating AMPK. Aging.

[8] Wang Y., Deng P., Liu Y., Wu Y., Chen Y., Guo Y., Zhang S., Zheng X., Zhou L., Liu W. (2020). Alpha-ketoglutarate ameliorates age-related osteoporosis via regulating histone methylations. Nat. Commun..

[9] Filip R.S., Pierzynowski S.G., Lindegard B., Wernerman J., Haratym-Maj A., Podgurniak M. (2007). Alpha-Ketoglutarate Decreases Serum Levels of C-terminal Cross-Linking Telopeptide of Type I Collagen (CTX) in Postmenopausal Women with Osteopenia: Six-Month Study. Int. J. Vitam. Nutr. Res..

[10] Boskey A., Coleman R. (2010). Aging and Bone. J. Dent. Res..

[11] Feng X., McDonald J.M. (2011). Disorders of Bone Remodeling. Annu. Rev. Pathol..

[12] Cui J., Shibata Y., Zhu T., Zhou J., Zhang J. (2022). Osteocytes in bone aging: Advances, challenges, and future perspectives. Ageing Res. Rev..

[13] Boyle W.J., Simonet W.S., Lacey D.L. (2003). Osteoclast differentiation and activation. Nature.

[14] Cedeno-Veloz B.A., Lopez J.E., Gutiérrez-Valencia M., Alegría L.L., Saiz L.C., García A.M.R., Latorre M.S., Vélez R.R., Izquierdo M., Martínez-Velilla N. (2022). Efficacy of Antiresorptive Treatment in Osteoporotic Older Adults: A Systematic Review and Meta-Analysis of Randomized Clinical Trials. J. Nutr. Health Aging.

[15] Zdzisińska B., Żurek A., Kandefer-Szerszeń M. (2017). Alpha-Ketoglutarate as a Molecule with Pleiotropic Activity: Well-Known and Novel Possibilities of Therapeutic Use. Arch. Immunol. Ther. Exp..

[16] Fraisl P., Aragonés J., Carmeliet P. (2009). Inhibition of oxygen sensors as a therapeutic strategy for ischaemic and inflammatory disease. Nat. Rev. Drug Discov..

[17] Chisolm D.A., Weinmann A.S. (2018). Metabolites, genome organization, and cellular differentiation gene programs. Curr. Opin. Immunol..

[18] Carey B.W., Finley L.W.S., Cross J., Allis C.D., Thompson C.B. (2015). Intracellular α-ketoglutarate maintains the pluripotency of embryonic stem cells. Nature.

[19] TeSlaa T., Chaikovsky A.C., Lipchina I., Escobar S.L., Hochedlinger K., Huang J., Graeber T., Braas D., Teitell M.A. (2016). α-Ketoglutarate Accelerates the Initial Differentiation of Primed Human Pluripotent Stem Cells. Cell Metab..

[20] Liu P.-S., Wang H., Li X., Chao T., Teav T., Christen S., Di Conza G., Cheng W.-C., Chou C.-H., Vavakova M. (2017). α-ketoglutarate orchestrates macrophage activation through metabolic and epigenetic reprogramming. Nat. Immunol..

[21] Hsu H., Lacey D.L., Dunstan C.R., Solovyev I., Colombero A., Timms E., Tan H.-L., Elliott G., Kelley M.J., Sarosi I. (1999). Tumor necrosis factor receptor family member RANK mediates osteoclast differentiation and activation induced by osteoprotegerin ligand. Proc. Natl. Acad. Sci. USA.

[22] Teitelbaum S.L. (2000). Bone Resorption by Osteoclasts. Science.

[23] Soysa N.S., Alles N., Shimokawa H., Jimi E., Aoki K., Ohya K. (2009). Inhibition of the classical NF-κB pathway prevents osteoclast bone-resorbing activity. J. Bone Miner. Metab..

[24] Galluzzi L. (2019). Heterogeneous cellular effects of α-ketoglutarate esters. Aging.

[25] Buddington R.K., Pajor A., Buddington K.K., Pierzynowski S. (2004). Absorption of α-ketoglutarate by the gastrointestinal tract of pigs. Comp. Biochem. Physiol. Part A Mol. Integr. Physiol..

[26] Wolffram S., Hagemann C., Grenacher B., Scharrer E. (1992). Characterization of the transport of tri- and dicarboxylates by pig intestinal brush-border membrane vesicles. Comp. Biochem. Physiol. Part A Physiol..

[27] Qi M., Liao S., Wang J., Deng Y., Zha A., Shao Y., Cui Z., Song T., Tang Y., Tan B. (2022). MyD88 deficiency ameliorates weight loss caused by intestinal oxidative injury in an autophagy-dependent mechanism. J. Cachexia Sarcopenia Muscle.

[28] Tang Y., Li J., Li F., Hu C.-A.A., Liao P., Tan K., Tan B., Xiong X., Liu G., Li T. (2015). Autophagy protects intestinal epithelial Cells against Deoxynivalenol toxicity by alleviating oxidative stress via IKK signaling pathway. Free. Radic. Biol. Med..

[29] Jiang Q., Tian J., Liu G., Yin Y., Yao K. (2019). Endoplasmic Reticulum Stress and Unfolded Protein Response Pathways Involved in the Health-Promoting Effects of Allicin on the Jejunum. J. Agric. Food Chem..

[30] Cherif H., Duhamel F., Cecyre B., Bouchard A., Quintal A., Chemtob S., Bouchard J.-F. (2018). Receptors of intermediates of carbohydrate metabolism, GPR91 and GPR99, mediate axon growth. PLoS Biol..

[31] Yuan Y., Xu P., Jiang Q., Cai X., Wang T., Peng W., Sun J., Zhu C., Zhang C., Yue D. (2020). Exercise-induced α-ketoglutaric acid stimulates muscle hypertrophy and fat loss through OXGR1-dependent adrenal activation. EMBO J..

[32] Xu C., Yuan Y., Zhang C., Zhou Y., Yang J., Yi H., Gyawali I., Lu J., Guo S., Ji Y. (2022). Smooth muscle AKG/OXGR1 signaling regulates epididymal fluid acid-base balance and sperm maturation. Life Metab..

[33] Indo Y., Takeshita S., Ishii K.-A., Hoshii T., Aburatani H., Hirao A., Ikeda K. (2013). Metabolic regulation of osteoclast differentiation and function. J. Bone Miner. Res..

[34] Fan J., Jahed V., Klavins K. (2021). Metabolomics in Bone Research. Metabolites.

[35] Dobrowolski P.J., Piersiak T., Surve V.V., Kruszewska D., Gawron A., Pacuska P., Håkanson R., Pierzynowski S.G. (2008). Dietary α-ketoglutarate reduces gastrectomy-evoked loss of calvaria and trabecular bone in female rats. Scand. J. Gastroenterol..

[36] Radzki R.P., Bienko M., Pierzynowski S.G. (2012). Anti-osteopenic effect of alpha-ketoglutarate sodium salt in ovariectomized rats. J. Bone Miner. Metab..

[37] Radzki R.P., Bieńko M., Filip R., Pierzynowski S.G. (2016). The protective and therapeutic effect of exclusive and combined treatment with alpha-ketoglutarate sodium salt and ipriflavone on bone loss in orchidectomized rats. J. Nutr. Health Aging.

[38] Tian Q., Zhao J., Yang Q., Wang B., DeAvila J.M., Zhu M.-J., Du M. (2020). Dietary alpha-ketoglutarate promotes beige adipogenesis and prevents obesity in middle-aged mice. Aging Cell.

[39] He W., Miao F.J.-P., Lin D.C.-H., Schwandner R.T., Wang Z., Gao J., Chen J.-L., Tian H., Ling L. (2004). Citric acid cycle intermediates as ligands for orphan G-protein-coupled receptors. Nature.

[40] Wright S.H., Hirayama B., Kaunitz J.D., Results T., Support T.F., Wright E.M. (2015). NaDC-1 (A-20): Sc-23539.

[41] Cummins E.P., Berra E., Comerford K.M., Ginouves A., Fitzgerald K.T., Seeballuck F., Godson C., Nielsen J.E., Moynagh P., Pouyssegur J. (2006). Prolyl hydroxylase-1 negatively regulates IκB kinase-β, giving insight into hypoxia-induced NFκB activity. Proc. Natl. Acad. Sci. USA.

[42] Takeda Y., Costa S., Delamarre E., Roncal C., De Oliveira R.L., Squadrito M.L., Finisguerra V., Deschoemaeker S., Bruyère F., Wenes M. (2011). Macrophage skewing by Phd2 haplodeficiency prevents ischaemia by inducing arteriogenesis. Nature.

[43] Harrison A.P., Pierzynowski S.G. (2008). Biological effects of 2-oxoglutarate with particular emphasis on the regulation of protein, mineral and lipid absorption/metabolism, muscle performance, kidney function, bone formation and cancerogenesis, all viewed from a healthy ageing perspective state of the art-review article. J. Physiol. Pharmacol. Off. J. Pol. Physiol. Soc..

