# Supplementary material for: Elevation of Intracellular Alpha-Ketoglutarate Levels Inhibits Osteoclastogenesis by Suppressing the NF-κB Signaling Pathway in a PHD1-Dependent Manner
Source: Nutrients. 2023 Jan 30;15(3):701. doi: 10.3390/nu15030701 (PMC9921543; doi:10.3390/nu15030701)
Supplement: Supplementary file 1 [file nutrients-15-00701-s001.zip › nutrients-2095603-supplementary.pdf]

Supplementary materials for:

The elevation of intracellular alpha-ketoglutarate levels inhibits osteoclastogenesis by suppressing the NF-κB signaling pathway in a PHD1-dependent manner

Junquan Tian [1, 2], Xuetai Bao [1, 2], Fan Yang [1, 2], Xiongzhuo Tang [3], Qian Jiang [1, 3*], Yuying Li [4], Kang Yao [1, 2*], Yulong Yin [1, 2, 3]

[1] Laboratory of Animal Nutritional Physiology and Metabolic Process, Key Laboratory of Agro-ecological Processes in Subtropical Region, National Engineering Laboratory for Pollution Control and Waste Utilization in Livestock and Poultry Production, Institute of Subtropical Agriculture, Chinese Academy of Sciences, Changsha, 410125, Hunan, China

[2] University of Chinese Academy of Sciences, Beijing, 100008, China

[3] College of Animal Science and Technology, Hunan Agricultural University, Changsha, 410000, Hunan, China

[4] Institute of Bast Fiber Crops, Chinese Academy of Agricultural Sciences, Changsha, 410205, Hunan, China

* Correspondence: jiangqian@hunau.edu.cn (Qian Jiang); yaokang@isa.ac.cn (Kang Yao)

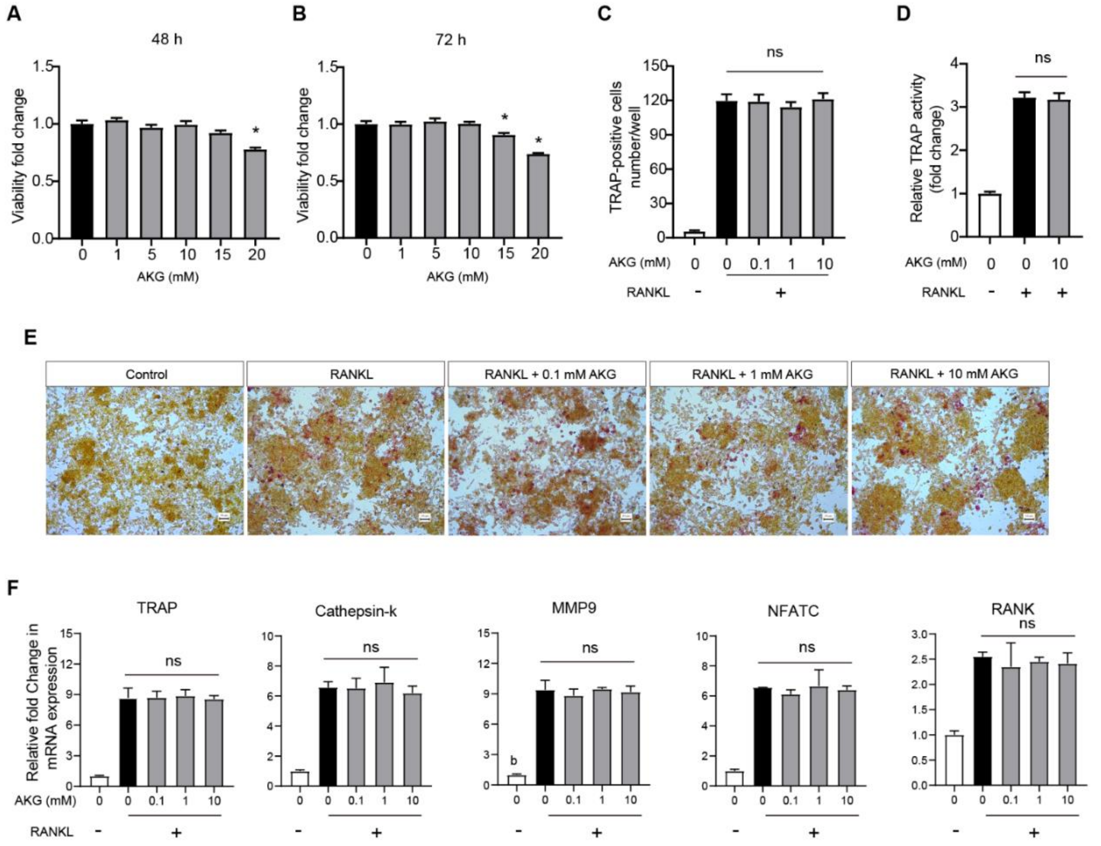

Figure S1. Extracellular AKG failed to inhibit RANKL-induced osteoclast differentiation in RAW264.7 cells.

(A-B) Effects of AKG on the viability of RAW264.7 cells (n=8). **(C)** Quantification of positive TRAP staining osteoclasts, **(D)** TRAP activity, **(E)** representative photographs of TRAP-positive cells, and **(F)** RT-qPCR analysis of mRNA levels of osteoclasts marker genes in RAW264.7 cells treated with RANKL (100 ng/ml) and DM-AKG for 3 days (n=3). Ns, no significance. *, $p < 0.05$.

Table S1. Primer sequence of the genes for RT-PCR in the study.

Gene	Species	Forward primer 5'-3'	Reverse primer 5'-3'
β-actin	Mouse	GGCTGTATTCCCCTCCATCG	CCAGTTGGTAACAATGCCATGT
TRAP	Mouse	CCAATGCCAAAGAGATCGCC	TCTGTGCAGAGACGTTGCCAAG
Cathepsin-k	Mouse	GTTGTATGTATAACGCCACGGC	CTTTCTCGTTCCCCACAGGA
MMP9	Mouse	CTGGACAGCCAGACACTAAAG	CTCGCGGCAAGTCTTCAGAG
NFATC	Mouse	CCGTTGCTTCCAGAAAATAACA	TGTGGGATGTGAACTCGGA
RANK	Mouse	CGAGGAAGATTCCCACAGAG	CAGTGAAGTCACAGCCCTCA
